# Presentation, management, and outcomes of central nervous system metastases in Africa: Systematic review and meta-analysis

**DOI:** 10.1093/noajnl/vdae219

**Published:** 2024-12-11

**Authors:** Sean O’Leary, W Elorm Yevudza, Peace Odiase, Muhammad Ammar Haider, Takara Newsome-Cuby, Odesanya Okikioluwa, Kwadwo Darko, Hannah Weiss, Umaru Barrie, Mabel Banson, Teddy Totimeh

**Affiliations:** Department of Neurosurgery, University of Texas Medical Branch, Galveston, Texas, USA; Department of Neurosurgery, Columbia University Irving Medical Center, New York, USA; Department of Biochemistry and Cancer Biology, Meharry Medical College, Nashville, Tennessee, USA; C.M.H. Lahore Medical College & Institute of Dentistry, Lahore, Pakistan; Kansas City University School of Osteopathic Medicine, Kansas City, Missouri, USA; All Saints University School of Medicine, Roseau, Dominica; Department of Neurosurgery, Korle-Bu Teaching Hospital, Accra, Ghana; Department of Neurosurgery, NYU Langone Health, New York, USA; Department of Neurosurgery, NYU Langone Health, New York, USA; Department of Neurosurgery, Korle-Bu Teaching Hospital, Accra, Ghana; Department of Neurosurgery, Accra Medical Centre, Accra, Ghana

**Keywords:** CNS, CNS metastases, central nervous system, global neurosurgery, LMICs, low- and middle-income countries, metastases

## Abstract

**Background:**

Central nervous system (CNS) metastases are a significant health challenge, particularly in Africa. This study evaluates the preclinical characteristics, primary causes, management strategies, and outcomes of CNS metastases in Africa.

**Methods:**

A systematic review of the literature was conducted using PubMed, Google Scholar, and Web of Science following PRISMA guidelines to identify studies on CNS metastases in Africa.

**Results:**

Thirty-one articles were reviewed, including 28 retrospective studies and 3 case reports. The retrospective studies comprised 12 552 patients, with 681 (5.42%) diagnosed with CNS metastases. Nigeria reported the highest number of cases (323), followed by Tunisia (180). The mean patient age was 48.20 years (range: 44.48-51.93), with a higher proportion in women (69.97%, 95% confidence interval [CI]: 54.59-85.35). Common symptoms were headaches (44.87%, 95% CI: 20.76-68.97) and motor deficits (21.39%, 95% CI: 6.40-36.38). Diagnostic tools included MRI (38.27%, 95% CI: 18.08-58.47) and CT (51.28%, 95% CI: 29.13-73.42). The most common primary tumor sites were breast (41.33%, 95% CI: 24.87-57.79) and lung (14.85%, 95% CI: 4.90-24.79). Treatment strategies involved surgery (62.01%, 95% CI: 33.01-91.01), radiotherapy (68.97%, 95% CI: 41.31-96.63), and chemotherapy (60.72%, 95% CI: 32.95-88.50). Outcomes included improved disease status in 34.99% (95% CI: 13.92-56.07), mortality in 44.88% (95% CI: 20.88-68.89), and loss to follow-up in 1.83% (95% CI: 0-3.72).

**Conclusion:**

CNS metastases in Africa show a higher proportion in women, with breast and lung cancers as the primary sources. Improved diagnostic and treatment strategies are essential to better patient outcomes.

**Key Points**

- Studies in Africa show CNS metastasis more prevalent in women than men.
- Breast and lung were the most common primary tumor sites in African patients.
- High mortality rates and significant loss to follow-up were common in studies.
- Improving healthcare resources and diagnostics is key to reducing CNS metastases.

Central nervous system (CNS) metastasis, involving the spread of malignant tumor cells from primary cancer sites to the brain and/or spinal cord, presents a significant healthcare challenge globally.^1–3^ The primary risk factors for developing CNS metastasis include the type and stage of the primary cancer, genetic predispositions, and access to timely and adequate health care.^2,4^ Breast, lung, and prostate cancers are the primary cancers most likely to metastasize to the CNS.^5,6^Breast cancer is a leading cause of CNS metastases.^5^ Up to 15% of breast cancers metastasize to the brain, with higher rates seen in patients with specific tumor subtypes and patients with genetic mutations such as *BRCA1/2* mutations.^7,8^ The rising incidence of these primary cancers has contributed to a higher prevalence of CNS metastases.^4^

The burden of CNS metastasis is especially pronounced in low- and middle-income countries such as those in Africa.^9,10^ In Africa, these challenges are especially compounded by a shortage of healthcare professionals in surgical subspecialties and limited opportunities for specialty training, including neurosurgery.^11^ Furthermore, these regions have seen the highest increases in cancer prevalence and mortality rates due to factors such as delayed diagnosis, limited access to advanced treatments, and challenges in obtaining high-quality neuro-oncological care.^4,12^ One significant hurdle in addressing CNS metastases in Africa is the delay in timely diagnosis and treatment.^13–15^ In addition, the limited access to advanced diagnostic tools including (MRI and CT) scans and inadequate healthcare facilities contribute to poorer outcomes for patients.^13–15^ For instance, in many regions, CNS involvement is often detected late in the disease course, negating the possibility of early intervention.^16^ Uwishema et al. reported that the incidence of brain metastasis in Nigeria is significantly underreported due to inadequate diagnostic facilities.^17^ Supporting this, Jedy-Agba et al. found that late-stage presentation of cancers, including those with potential CNS involvement, is a widespread issue.^18^ Their studies on breast cancer in sub-Saharan Africa highlight systemic problems such as limited access to pathology services, diagnostic tools, and trained healthcare professionals.^18^ These deficits result in many cases of metastases remaining undetected until they reach an advanced stage, significantly impacting patient outcomes.

In this study, we review existing literature on CNS metastases in Africa to estimate its proportion, characterize the primary etiologies of cancer, evaluate demographic and clinical features, assess treatment strategies and effectiveness, and analyze long-term patient outcomes. Our study aims to describe the existing heterogeneous literature across Africa and identify areas for future research.

## Methods

### Search Strategy

**Figure 1.**
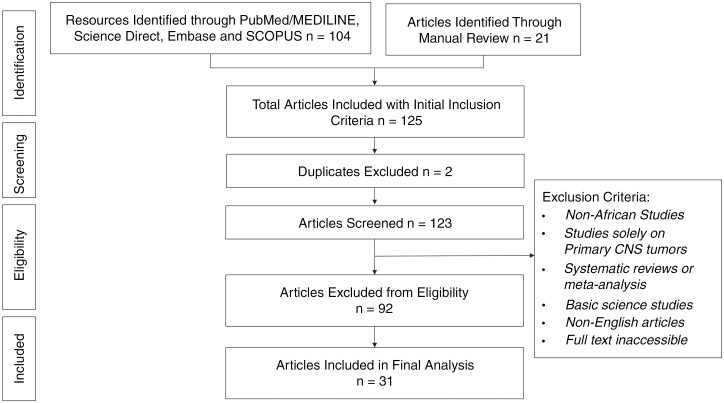
PRISMA flow diagram showcasing the methodology employed in conducting the systemic review.

The search methodology followed the Preferred Reporting Items for Systematic Reviews and Meta-analyses (PRISMA) guidelines examining medical literature from PubMed/MEDLINE, SCOPUS, Embase, Google Scholar, and Science Direct databases (see Figure 1). This study was not registered in the International Prospective Register of Systematic Reviews registry. We used the Boolean search: (“Central Nervous System Metastasis” OR “Brain Metastasis” OR “Spinal Cord Metastasis” OR “CNS Metastasis” OR “Metastatic Brain Tumor” OR “Metastatic Spinal Cord Tumor” OR “Metastatic Lesions in Brain” OR “Metastatic Lesions in Spinal Cord”) AND “Africa” AND (“Presentation” OR “Symptoms” OR “Clinical Presentation” OR “Diagnosis” OR “Evaluation” OR “Assessment” OR “Management” OR “Treatment” OR “Therapeutics” OR “Intervention” OR “Care” OR “Outcome” OR “Prognosis” OR “Survival” OR “Mortality” OR “Quality of Life”). There was no time frame limit to the search strategy. Retrospective, prospective, case reports, case series, comparative studies, and clinical trials that discussed metastatic tumors to the brain and/or spinal cord in both adult and pediatric patients in Africa were included in the final analysis. The exclusion criteria for our analysis were studies lacking original data (such as systematic reviews and meta-analyses), and articles not published in English.

### Data Extraction

Five authors (S.O., E.Y., M.A.H., T.N.C., and O.O.) independently conducted database searches, followed by title, abstract, and full-text reviews, to determine inclusion based on the predetermined criteria. Bibliographies of included articles were manually reviewed to identify additional articles that might have been missed in the original search. The following variables were extracted from the included articles: demographics of both CNS and non-CNS metastasis patients, imaging modalities, histopathological diagnoses, primary tumor location, locations of metastasis to the CNS, treatment modalities, management complications, and outcome at last follow-up.

### Statistical Analysis

Proportional meta-analyses were carried out in R Studio (Version 4.3) using the *metafor, meta, and metadata* packages. This analysis pooled data to estimate the proportion of CNS metastases. Subgroup analysis based on regions within Africa (North, East, West, and South) with countries classified using the UN geoscheme for Africa^19^ was conducted to investigate potential variations in reported proportions across these different regions. Given the expected high variability among the individual studies, data aggregation was achieved using a random effects model with precision evaluated by 95% confidence intervals (95% CI). The Cochran Q statistic was employed to evaluate the heterogeneity across studies, whereas the *I*² test was utilized to measure the extent of this heterogeneity. In addition, our approach to summarizing categorical data involved computing frequencies and percentages for each category, derived from individual studies. For visual representation, proportions were depicted in forest plots.

## Results

### Electronic Search Yield

Initially, 125 sources were identified following the described PRISMA methodology (see Figure 1). After eliminating 2 duplicate sources and evaluating 123 sources against our inclusion and exclusion criteria, we included 31 articles (28 retrospective articles detailed in Supplemental Table 1, and 3 case reports detailed in Supplemental Table 2) from 10 African countries (Cameroon, Ethiopia, Ivory Coast, Kenya, Nigeria, Senegal, South Africa, Tunisia, Uganda, and Zimbabwe). The retrospective studies were analyzed and included 12 552 patients, with 681 patients (5.4%) diagnosed with CNS metastasis.

**Figure 2.**
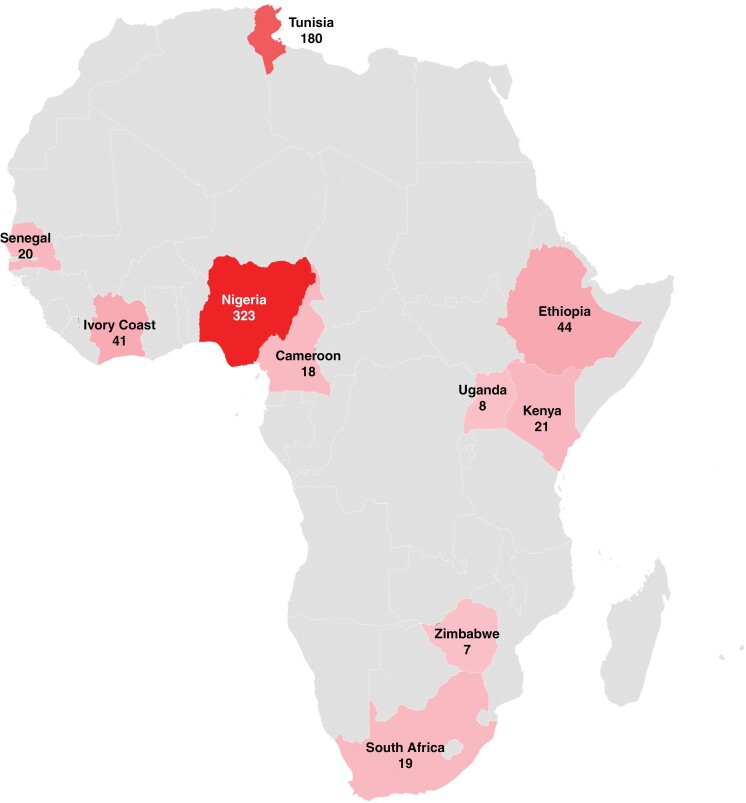
African map showing the distribution of cumulative cases of CNS metastases in the included articles.

West Africa (Cameroon, Ivory Coast, Nigeria, and Senegal) contributed the majority of patients—59.0% (402/681)—with CNS metastasis, whereas North Africa (Tunisia) contributed 26.43% (180/681) patients, East Africa (Ethiopia, Kenya, and Uganda) contributed 10.72% (73/681) patients, and Southern Africa (Zimbabwe and South Africa) added 3.82% (26/681) patients (Table 1). Nigeria had the highest number of published studies at 13 (46.2%), followed by South Africa with 5 studies (17.9%). Nigeria also had the largest number of CNS metastasis patient cohort with 47.43% (323/681) patients, followed by Tunisia and Ethiopia with 26.43% (180/681) and 6.46% (44/681) patients, respectively (Figure 2).

**Table 1.** Cumulative Frequency of 12 552 CNS and Non-CNS Metastatic Tumor Patients Stratified by Country in the 28 Retrospective Studies

Region	Country	Total number of patients	CNS Mets	No CNS Mets	Number of articles
West Africa	Nigeria	1800	17.94% (323)	82.06% (1477)	13
	Cameroon	260	6.92% (18)	93.08% (242)	1
	Cote D’Ivoire	41	100% (41)	0 (0)	1
	Senegal	682	2.93% (20)	97.07% (662)	1
East Africa	Uganda	172	4.65% (8)	95.35% (164)	1
	Ethiopia	217	20.27% (44)	79.72% (173)	1
	Kenya	256	8.2% (21)	91.8% (235)	2
Southern Africa	Zimbabwe	351	1.99% (7)	98.01% (344)	1
	South Africa	318	5.98% (19)	94.03% (299)	5
North Africa	Tunisia	8455	2.13% (180)	97.87% (8275)	2
**Total**		12 552	5.43% (681)	94.57% (11 871)	28

### Demographics, Clinical Characteristics, Diagnostics, Management Strategies, and Outcomes of Retrospective Studies

From the 28 retrospective studies, data on 12 552 patients (Table 2) with 681 (5.42%) CNS metastatic disease were analyzed (Table 3). The mean age of these patients was 48.2 years (95% CI: 44.48-51.93). Gender data reported in 15 articles indicated 71.84% were female (301/419). The most common symptoms were headaches in 44.87% of patients (95% CI: 20.76-68.97, 10 articles), motor deficits in 21.39% (95% CI: 6.40-36.38, 10 articles), and seizures in 12.25% (95% CI: 2.71-21.79, 10 articles). Diagnostic imaging predominantly used CT, with a proportion of 51.28% (95% CI: 29.13-73.42, 15 articles), and MRI, with a proportion of 38.27% (95% CI: 18.08-58.47, 15 articles). Autopsies confirmed CNS metastatic lesions in 21.29% of cases (95% CI: 2.56-40.01, 15 articles). Breast cancer was the most common primary tumor, accounting for 41.33% (95% CI: 24.87-57.79, 24 articles), followed by lung cancer at 14.85% (95% CI: 4.90-24.79, 24 articles). Details on primary tumor locations are further outlined in Table 3.

**Table 2.** Descriptive Summary and Meta-analyses of 12 552 patients with CNS and non-CNS Metastases in the 28 Retrospective Studies

Demographics	Descriptive summary		Pooled prevalence/means		
	Sum of patients/number of reported patients (%)	Number of articles	Prevalence (%)	CI.LB-UB	*I* ^2^
Male	832/2717 (30.62%)	19	36.59	23.67-49.52	99.76
Female	1332/2717 (49.02%)	19	54.70	41.24-68.15	99.64
*Age (years): 44.88 (95% CI: 37.96-51.80)*					
**Primary tumor**					
Retinoblastoma	217/12511 (1.73%)	27	4.35	0.00-11.58	99.99
Breast cancer	2427/12511 (19.40%)	27	27.85	11.51-44.18	100.00
GTD	78/12511 (0.62%)	27	4.31	0-11.51	99.99
Melanoma	15/12511 (0.12%)	27	3.77	0-10.15	99.99
Lung cancer	213/12511 (1.70%)	27	4.88	0-12.14	99.98
Prostate cancer	73/12511 (0.58%)	27	4.34	0-11.53	99.99
Colorectal cancer	8/12511 (0.06%)	27	0.10	0.04-0.15	0.00
Primary brain cancer	1838/12511 (14.69%)	27	35.51	19.23-51.79	100.00
Spinal cord	17/12511 (0.14%)	27	0.04	0-0.09	3.75
**Metastatic tumor locations**					
Vertebral column	34/12552 (0.27%)	28	0.04	0-0.10	3.92
Chest wall	73/12552 (0.58%)	28	0.01	0-0.03	0.00
Long bones	284/12552 (2.26%)	28	5.30	0.93-9.67	99.94
Liver	91/12552 (0.72%)	28	0.01	0-0.03	0.00
Lungs	191/12552 (1.52%)	28	3.68	0.81-6.55	99.89
Lymph nodes	67/12552 (0.53%)	28	5.01	0-11.94	99.99
Scalp	1/12552 (0.01%)	28	0.04	0-0.08	3.36
Gastrointestinal tract	1/12552 (0.01%)	28	0.04	0-0.08	3.36
Abdominal viscera	432/12552 (3.34%)	28	7.35	1.05-13.66	99.96
Brain^a^	676/678 (99.71%)	27	99.96	99.27-100	0.00
Spinal cord^a^	2/678 (0.29%)	27	0.04	0.29-2.00	0.00
**Primary tumor management**					
Surgical management	513/2116 (24.24%)	8	35.10	12.47-57.73	99.84
Radiation	76/143 (53.15%)	3	55.21	3.32-100	99.30
Chemotherapy	506/668 (75.75%)	5	70.67	45.56-95.77	98.87
Hormonal therapy	278/629 (44.20%)	5	40.93	23.16-58.70	95.57
**Secondary tumor management**					
Surgical management	350/1581 (22.14%)	3	10.40	0-24.60	98.47
Radiation	452/2158 (20.95%)	6	49.14	16.67-81.61	99.85
Chemotherapy	343/1531(22.40%)	2	40.78	0-84.67	99.06

Abbreviations: CI.LB-UB, confidence interval lower bound to upper bound; CNS, central nervous system; *I*^2^, percentage of variation due to heterogeneity; Mets, metastases; GTD, gestational trophoblastic disease.

^a^One article did not describe a location within the CNS and thus was excluded here.

**Table 3.** Descriptive Summary and Meta-analyses of 678 Patients with CNS Metastases in the 28 Retrospective Studies

Demographics	Descriptive summary		Pooled prevalence/means		
	Sum of patients/number of reported patients (%)	Number of articles	Prevalence (%)	CI.LB-UB	*I* ^2^
Age	401/401 (100%)	7	48.20	44.48-51.93	83.51
Male	118/419 (28.16%)	15	30.03	14.65-45.41	98.51
Female	301/419 (71.84%)	15	69.97	54.59-85.35	98.51
**Signs and symptoms**					
Headaches	152/350 (43.43%)	10	44.87	20.76-68.97	99.01
Motor deficit	78/350 (22.29%)	10	21.39	6.40-36.38	97.15
Seizures	46/350 (13.14%)	10	12.25	2.71-21.79	94.27
Intracranial hypertension	21/350 (6.00%)	10	8.51	0-18.79	98.22
Altered mental status	19/350 (5.43%)	10	5.87	0.57-11.16	86.41
Visual deficits	17/350 (4.86%)	10	5.72	0-11.82	93.53
Cerebellar syndrome	15/350 (4.29%)	10	4.81	0.65-8.98	77.19
Nausea	16/350 (4.57%)	10	1.11	0-2.35	9.71
Vomiting	14/350 (4.00%)	10	1.03	0-2.20	7.59
Cranial nerve palsy	6/350 (1.71%)	10	0.72	0-1.60	0.44
Memory disorder	12/350 (3.43%)	10	0.70	0-1.55	0.00
**Diagnostic modality**					
MRI	92/416 (22.12%)	15	38.27	18.08-58.47	98.69
CT	157/416 (37.74%)	15	51.28	29.13-73.42	99.45
Mammogram	48/416 (11.54%)	15	18.26	0.65-35.86	99.60
Autopsy	59/416 (14.18%)	15	21.29	2.56-40.01	99.65
**Location of primary tumor**					
Retina	46/633 (7.27%)	24	8.82	0-17.82	98.97
Parotid gland	1/633 (0.16%)	24	0.90	0.18-1.61	0.00
Thyroid gland	5/633 (0.79%)	24	1.21	0.32-2.10	3.72
Ovary	2/633 (0.32%)	24	0.98	0.21-1.74	0.94
Pancreas	3/633 (0.47%)	24	0.98	0.21-1.75	1.01
Liver	3/633 (0.47%)	24	1.03	0.24-1.83	1.62
Prostate	8/633 (1.26%)	24	0.99	0.27-1.70	0.00
Uterus	38/633 (6.00%)	24	9.20	2.41-15.98	97.67
Gastrointestinal tract	9/633 (1.42%)	24	1.67	0.70-2.65	0.00
Breast	275/633 (43.44%)	24	41.33	24.87-57.79	99.22
Lungs	117/633 (18.48%)	24	14.85	4.90-24.79	98.03
Esophagus	1/633 (0.16%)	24	0.89	0.18-1.61	0.00
Kidneys	4/633 (0.63%)	24	1.08	0.26-1.91	2.27
Adrenal gland	6/633 (0.95%)	24	1.00	0.23-1.78	1.36
Lymph nodes	10/633 (1.58%)	24	7.71	0.10-15.32	98.61
Skin	6/633 (0.95%)	24	1.52	0.59-2.45	0.34
**Location of metastasis**					
Brain	676/681 (99.27%)	28	98.74	97.90-99.58	4.34
Spinal cord	2/681 (0.29%)	28	0.91	0.21-1.60	0.00
**Treatment**					
Surgical management	84/232 (36.21%)	8	62.01	33.01-91.01	98.15
Nonsurgical management					
Steroids	47/238 (19.75%)	4	47.89	0-101.85	99.94
Anticonvulsants	41/238 (17.23%)	4	26.99	0-74.47	99.92
Hormonal therapy	11/59 (18.64%)	2	18.57	8.65-28.48	0.00
Whole brain radiotherapy	288/367 (78.47%)	8	68.97	41.31-96.63	99.60
Chemotherapy	59/109 (54.12%)	4	60.72	32.95-88.50	91.26
**Outcomes**					
Improved diseasea	80/179 (44.69%)	8	34.99	13.92-56.07	92.73
Death	52/179 (29.05%)	8	44.88	20.88-68.89	98.55
Lost to follow-up	7/179 (3.91%)	8	1.83	0-3.72	0.00

Abbreviations: CI.LB-UB, confidence interval lower bound to upper bound; CNS, central nervous system; ER, estrogen receptor; HER2, human epidermal growth factor receptor 2; *I*^2^, percentage of variation due to heterogeneity; Mets, metastases; PR, progesterone receptor.

^a^Improvement in disease is defined as a decrease in metastatic burden, an improvement in clinical symptoms, or remission from metastatic disease.

Surgical management was reported in 62.01% of cases (95% CI: 33.01-91.01, 8 articles), including procedures such as excision biopsy and total resection. Radiation therapy was reported in 68.97% (95% CI: 41.31-96.63, 8 articles) and chemotherapy in 60.72% (95% CI: 32.95-88.50, 4 articles) of patients. Nonsurgical treatments included steroids for 47.89% (95% CI: 0-101.85, 4 articles) and anticonvulsants for 26.99% (95% CI: 0-74.47, 4 articles). Postoperative complications were not reported in any of the included studies. Mortality data, reported in 8 articles, showed a proportion of 44.88% (95% CI: 20.88-68.89) among CNS metastatic patients.

### Comparative Meta-analysis of Proportion of CNS Metastasis in Africa by Region

**Figure 3.**
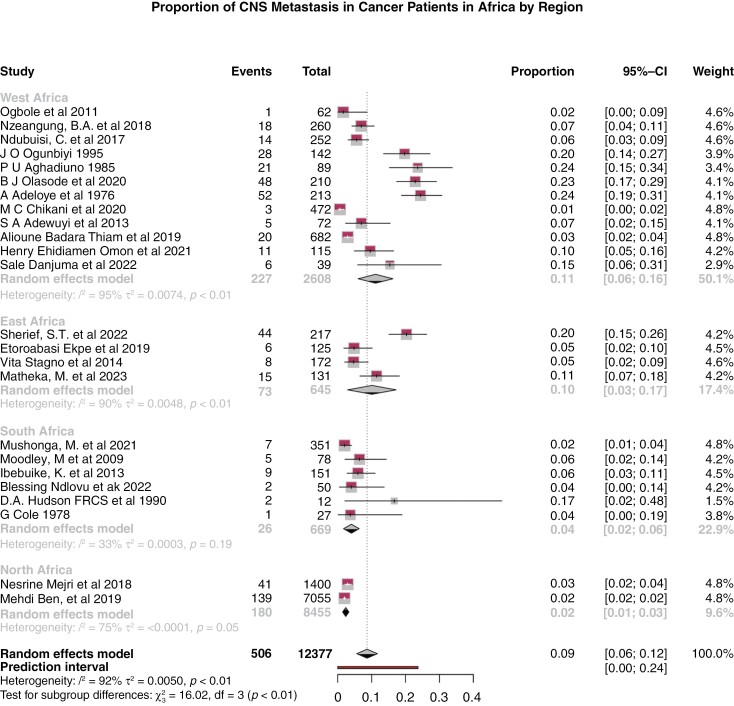
Forest plot depicting the proportion of CNS metastasis, stratified by different regions of Africa. Studies in which the sum of the events equaled the total number of patients (studies exclusively focusing on patients with CNS metastases) were excluded from the regional comparative analysis.

Figure 3 shows the proportion of CNS metastases among all patients with metastatic disease based on existing published literature. The overall estimated proportion across all regions was 9.0% (95% CI: 6.0-12.0%, *P* < .01). Regional variations are noted, with West Africa reporting the highest proportion and North Africa the lowest. In West Africa, proportions ranged from 0.01 to 0.24 across 12 studies, with a pooled proportion of 11.0% (95% CI: 6.0-16.0%, *P* < .01). East Africa showed proportions ranging from 0.02 to 0.20 across 7 studies, with a pooled proportion of 10.0% (95% CI: 3.0-17.0%, *P* < .01). South Africa, from 6 studies, reported proportions from 0.02 to 0.17, with a pooled proportion of 4.0% (95% CI: 2.0-6.0%, *P* < .01). North Africa, based on 2 studies, reported very low proportions with a pooled proportion of 2.0% (95% CI: 1.0-3.0%, *P* < .01).

## Discussion

Our systematic review and meta-analysis revealed that CNS metastases reported in the literature in Africa predominantly affect middle-aged adults, especially women, with a high proportion of primary breast and lung cancer. Primary breast cancer accounted for 43.44% of CNS metastasis cases, followed by lung cancer at 18.48%. These results align with the known global patterns of cancer spread to the CNS, which disproportionately impact individuals with breast and lung cancers.^20^ Notably, the high mortality rate and significant number of cases lost to follow-up highlight substantial gaps in the diagnostic and treatment capabilities across the continent. Limited resources and inadequate healthcare infrastructure contribute to delayed diagnoses and suboptimal management of CNS metastases.^21,22^ Furthermore, the absence of standardized treatment protocols and access to advanced therapeutic options exacerbates the situation, leading to poorer outcomes for patients.^23^ This is evidenced by the fact that a large number of cases reviewed were diagnosed and documented at autopsy rather than during clinical management, with autopsies confirming CNS metastatic lesions in 21.29% of the cases. Moreover, the limited data availability, as indicated by the small number of countries represented in the literature, presents a major challenge in accurately assessing and managing CNS metastatic prevalence across Africa.

The significant variance in the availability of diagnostic modalities, such as MRI and CT scans, observed across different African regions, suggests a disparity in healthcare resources that is likely contributing to delayed diagnoses and suboptimal treatment outcomes. MRI was utilized in 22.12% of patients, whereas CT scans were used in 37.74%. The importance of early detection and effective management of metastatic disease is critical.^23^ Early intervention improves patient prognoses and reduces healthcare expenditures by decreasing the likelihood of costly hospitalizations and extended care needs.^24^ CT scans have been shown to be less expensive, and highly effective in localizing metastatic tumors, providing a critical alternative to more expensive MRI.^22^ The cost of diagnostics is still out of reach of most sections of the population. A nonuniform approach to procurement of these machines means that the price of machines ranges widely across the continent with reflection in the cost of exams. In Ghana, an MRI with contrast costs around 100 USD, whereas in Zimbabwe, the same procedure can cost as much as 450 USD.^25^ The continental societies must integrate health and cancer care into their policy discussions in order to bridge these gaps.

The absence of comprehensive brain tumor registries and reliable data reporting in Africa, especially in sub-Saharan African countries, makes the assessment of the burden of CNS metastases very difficult. Consequently, estimates of disease proportion are often based on reported cases and retrospective cohort studies from individual countries, which can lead to misconceptions about the incidence rates of certain brain tumors.^26^ This is in contrast to observations made by neurosurgeons working in Africa, who have noted higher incidences than what is reflected in the limited data.^27,28^ In this regard, our study, constrained by the limited number of studies and including only 10 out of 54 African countries, revealed a notably high proportion of CNS metastases in Nigeria, Tunisia, and Ethiopia. Specifically, Nigeria stands out as a major contributor, with 47.4% of the total reported cases, encompassing 323 patients. Similarly, Tunisia and Ethiopia also show significant numbers, but the data for these countries are less extensive. It is very likely that the high proportion in Nigeria is influenced by reporting bias rather than reflecting the true picture of CNS metastases.

Variations in data reporting and publication practices could skew the observed figures, potentially exaggerating the actual burden in Nigeria compared with other regions. For example, the pioneering efforts of faculty and local and regional collaborations in training and research development have significantly contributed to the growth of research in Nigeria.^29^ These disparities may also be explained by the scope of the included studies, with Nigeria representing the largest number of studies and patients with CNS metastases.^30–42^ This is also influenced by the research output of the respective countries. Four of the included Nigerian articles focused solely on the management of patients with documented CNS metastases. Tunisia, although only contributing data from 1 study, conducted a retrospective analysis of 1400 breast cancer patients treated over a 10-year period at 2 institutions, gathering a large amount of data including metastatic CNS cancers.^43^ These differences in regional CNS metastasis could also stem from disparities in the availability of diagnostic tools and treatment facilities, which can influence the detection rates and outcomes.^44^ These disparities can also extend to the research output of individual countries, affecting the overall understanding and management of CNS metastases in various regions.

South Africa contributed the second highest number of articles (*n* = 5), but these studies represented only 318 patients (2.53% of total CNS and non-CNS metastatic cases) and 180 patients (2.13% of CNS metastatic cases). This suggests that despite a strong academic output, the patient data captured may be limited, likely due to the concentration of research in urban centers with better resources but lower population coverage. Supporting this, the articles were produced by academic centers in just 3 of the 9 South African provinces, with the majority (60%, 3/5) originating from Johannesburg in Gauteng Province,^45–47^ 1 from Cape Town in the Western Cape Province,^3^ and 1 in KwaZulu-Natal.^48^ Notably, there were no studies from 6 of the 9 provinces, including the Eastern Cape, Free State, Limpopo, Mpumalanga, Northern Cape, and North West Province. This geographic imbalance highlights a microcosm of the need for broader, more inclusive research efforts across the entire country to ensure a more representative understanding of CNS metastases.

Regional variations across West, East, Southern, and North Africa must be carefully considered when interpreting data on CNS metastases. North Africa, for example, benefits from socialized healthcare systems that facilitate comprehensive data collection.^49^ In our study, Tunisia, the only North African country represented, contributed data on 97.85% of non-CNS metastases but only 2.13% of CNS metastasis cases.^43^ The equitable access to health care in this region likely ensures that the reported data more accurately reflect the general population. In contrast, West Africa, which accounted for over 59% of the CNS metastasis cases,^30–42,50–52^ primarily relies on an out-of-pocket payment system.^53^ Consequently, the data from West Africa may not represent the broader population, as they likely reflect only those who can afford medical care. Expanding data collection efforts in North Africa to encompass CNS metastases would significantly enhance research aimed at addressing the needs within these countries. Meanwhile, establishing state-funded registries or privately funded initiatives is crucial to accurately assess the burden of CNS metastatic disease across sub-Saharan Africa.

This regional disparity highlights the critical need for more comprehensive data collection and the development of robust brain tumor registries across Africa. Improved data collection would help in accurately assessing the true burden of CNS metastases and addressing the regional disparities in diagnosis and treatment.^54^ Such efforts are essential for developing targeted strategies to better understand and, in turn, manage and reduce the impact of CNS metastases throughout the continent.

Despite efforts toward surgical management and radiotherapy, with usage in 62.01% and 68.97% of patients, respectively, the high mortality rates underscore the urgent need for treatment strategies tailored to the resource constraints of many African healthcare settings. In high-income nations, the availability of radiotherapy machines is significantly higher, with one machine for every 120 000 people.^55^ Contrastingly, in middle-income countries, a single machine may serve more than 1 million individuals.^56^ This disparity grows in low-income countries, where 1 machine often supports over 5 million people, as observed in several African nations including Nigeria, Ethiopia, and the Democratic Republic of Congo.^56^ Furthermore, 51 countries worldwide, including about half from Africa, completely lack access to radiotherapy treatments.^56^

In our study, metastatic breast cancer accounted for 43.44% of CNS metastasis cases in Africa. Previous research in the United States and Europe, however, indicates that brain metastases primarily originate from lung cancer at rates of 39% to 50%, followed by breast cancer at 15% to 30%, and melanoma at 6% to 11%.^57^ The relatively high percentage of breast cancer resulting in CNS metastasis observed in our data may be attributed to the higher prevalence of aggressive breast cancer subtypes, such as triple-negative and HER2-positive tumors, and late-stage presentation, which are more frequently seen among women of African descent.^58–60^ Additionally, the generally better prognosis of breast cancer compared with lung cancer subtypes may contribute to the higher observed rates of CNS metastasis due to a survivorship bias. However, the increased rate of breast cancer metastases may be a secondary reporting bias rather than reflective of current disease patterns. This finding is noteworthy, as certain primary breast cancers (HER2-positive, ER/PR-positive, and TNBC) and lung cancers (ALK, ROS1, and EGFR mutations) with actionable mutations show strong intracranial response rates to targeted therapies.^61,62^ Consequently, ensuring access to targeted therapies for these primary cancers may extend to managing their associated CNS metastases, emphasizing the role of interdisciplinary collaboration in improving outcomes.^63^

The high mortality rate among relatively young individuals with CNS metastatic disease on the world’s youngest continent has far-reaching implications, as Africa is expected to be a key human resource reservoir in the future, particularly as other regions face aging populations.^64^ Therefore, global efforts should be viewed not merely as altruistic outreach but as pragmatic interventions critical to safeguarding the future. Continental advocacy organizations like the Society for Neurooncology Sub-Saharan Africa (SNOSSA) and the Brain Tumor Consortium of Africa (BTCA) make for powerful platforms to launch registries, collaborative studies and trials, donation of equipment and consumables, and training opportunities for medical staff.^63,65^ Such organizations should be helped at governmental levels to unify voices in cancer care on the continent.

### Limitations

This systematic review and meta-analysis consolidate evidence regarding metastatic CNS tumors in Africa. The data, derived solely from hospital settings, may not fully capture the epidemiology of metastatic brain tumors across the continent, given the substantial barriers to healthcare access and publications. However, these findings offer a glimpse into adult neuro-oncology practices in Africa and underscore the importance of focusing on population-based research.

While systematic reviews and meta-analyses are crucial for summarizing existing literature, they also present challenges in comparing studies with varied designs and methodologies. This difficulty is particularly pronounced when studies use diverse outcome measures. We observed variability in the reporting of adjuvant therapies, patient performance statuses, and mortality rates, which raises concerns about publication bias. These issues diminish the quality of evidence and restrict the reach of our conclusions.

Despite English being the primary language of scientific publication worldwide, many nations conduct research in their official local languages, such as Arabic, Portuguese, or Spanish.^66–68^ Our analysis was limited to English publications due to our team’s linguistic capabilities. This decision inevitably excluded many relevant studies, potentially omitting critical data. Notably, we disregarded a considerable amount of research on bony metastases to the vertebral column, a significant topic in our preliminary search, to maintain the integrity of our findings.

Moreover, the limited number of countries represented in the studies included in this review presents a significant limitation. Only 10 out of 54 African countries were included, with a heavy concentration of data from Nigeria, which had the highest number of studies and patients with CNS metastases. This geographic skew can lead to an overrepresentation of the proportion and characteristics of CNS metastases in Nigeria while underrepresenting other regions with different healthcare contexts and challenges. Additionally, most of the included studies are retrospective, which can introduce biases related to data collection and reporting practices.

Another limitation of this review is the absence of molecular characterization of tumors across the continent, which is particularly important given the resource constraints in many African countries. Accurate molecular diagnosis is essential for optimizing the use of limited resources, allowing for more targeted and effective treatment strategies.^69^ To address this, the establishment of centers of excellence, which can concentrate expertise and resources in strategic regions, could serve as a key interim solution. These centers would not only improve diagnostic accuracy but also enhance access to care for at-risk populations. Moreover, fostering such centers could facilitate the development of regional networks for research and training, ultimately contributing to a more comprehensive understanding and management of CNS metastases across Africa.

## Conclusion

This study summarizes existing literature on CNS metastatic disease and demonstrates that, according to current published African literature, CNS metastases in Africa predominantly arise from primary breast and lung cancers in middle-aged adults. High mortality and substantial loss to follow-up highlight significant deficiencies in healthcare infrastructure, diagnostic capabilities, and treatment accessibility in Africa. Enhancing healthcare resources, early diagnostic methods, treatment strategies, and research is essential to mitigate the impact of CNS metastases and improve patient outcomes.

## Supplementary Material

vdae219_suppl_Supplementary_Table_S1

vdae219_suppl_Supplementary_Table_S2
