# Supplementary material for: Presentation, management, and outcomes of central nervous system metastases in Africa: Systematic review and meta-analysis
Source: Neurooncol Adv. 2024 Dec 11;7(1):vdae219. doi: 10.1093/noajnl/vdae219 (PMC11805695; doi:10.1093/noajnl/vdae219)
Supplement: vdae219_suppl_Supplementary_Table_S1 [file vdae219_suppl_supplementary_table_s1.docx]

**Supplemental Table 1.** Demographics, pre-clinical characteristics, management strategies and outcomes of retrospective and comparative studies included in the systematic review.

| **#** | **Author, Year, Country** | **No. of Patients with CNS Metastasis/Total No. of Patients** | **Mean Age (Years) ± SD, (Range)** | **Sex** | **Presenting Signs and Symptoms (#)** | **Imaging Modality (#)** | **Diagnostic Findings (#)** | **Pathological Findings (#)** | **Primary**  **Cancer (#)** | **CNS Metastasis Locations (#)** | **Management; Complications** | **Mortality; Overall Survival (Months)** |
| --- | --- | --- | --- | --- | --- | --- | --- | --- | --- | --- | --- | --- |
| 1 | *Ogbole GI et al. 2011, Nigeria*^21^ | 1/62 | NA | NA | Visual Deficits (1), Headaches (1), Paraplegia (1) | MRI (1) | Parasellar Mass (1) | Thyroid Carcinoma (1) | Thyroid Gland (1) | Brain (1):  Supratentorial (1), Leptomeninges (1), Cerebrum (1) | NA;NA | NA; NA |
| 2 | *Sherief ST et al, 2022, Ethiopia*^26^ | 44/217 | NA | NA | NA | CT (NA), MRI (NA) | CNS Metastatic Lesions (44) | Retinoblastoma (44) | Eye (44) | Brain (44) | NA;NA | NA; NA |
| 3 | *Adegboyega B et al, 2023, Nigeria*^1^ | 52/52 | 53 ± 12.3 [NA] | 9 M, 43 F | NA | NA | NA | Anaplastic thyroid (1), Unspecified Breast Cancer (34), Carcinoid Tumor (1), Unspecified Lung Cancer (1), Unspecified Parotid Cancer (1), Unspecified Prostate Cancer (1), Unspecified Renal Cancer (14) | Thyroid (1), Breast (34), GI (1), Head and neck (3), Thigh (1), Lungs (7), Parotid (1), Prostate (1), Renal (1), Others (2) | Brain (52) | RT (52); NA | 32; Median OS: 6 |
| 4 | *Nzeangung BA et al, 2018, Cameroon*^20^ | 18/260 | NA | NA | NA | NA | NA | Unspecified Breast Cancer (18) | Breast (18) | Brain (18) | NA;NA | NA; NA |
| 5 | *Ndubuisi CA et al, 2017, Nigeria*^19^ | 14/252 | NA ± NA [21-90] | 3 M ,11 F | NA | CT (NA), MRI (NA) | NA | NA | NA | Brain (14): Infratentorial (2) | NA;NA | NA; NA |
| 6 | *Mushonga M et al, 2021, Zimbawe*^17^ | 7/351 | NA | NA | NA | Mammogram (7), Surgical Biopsy (7) | CNS Metastatic Lesions (7) | Breast Carcinoma (7) | Breast (7) | Brain (7) | RT (7); NA | NA; NA |
| 7 | *Moodley M et al, 2009, South Africa*^16^ | 5/78 | 31 ± NA [16-50] | NA | NA | CT (NA), MRI (NA) | CNS Metastatic Lesions (5) | Choriocarcinoma (5) | Uterus (5) | Brain (5): Supratentorial (5) | NA;NA | 3; NA |
| 8 | *Ibebuike K et al, 2013, South Africa*^11^ | 9/151 | NA | NA | NA | CT (9), MRI (9) | CNS Metastatic Lesions (9) | NA | Head/Neck (9) | Brain (9) | NA;NA | 3; NA |
| 9 | *Ndlovu B et al, 2022, South Africa^18^* | 2/50 | NA | NA | NA | NA | NA | NA | NA | Brain (2) | Surgery (2); NA | NA; NA |
| 10 | *Hudson DA et al, 1990, South Africa*^10^ | 2/12 | 55.5 ± NA [NA- NA] | NA | NA | NA | CNS Metastatic Lesions (2) | NA | Head/Neck (2), External Ear (2) | Brain (2) | NA;NA | 2; OS 19-37 |
| 11 | *Ogunbiyi JO et al, 1995, Nigeria*^22^ | 28/142 | NA | NA | NA | Autopsy (28) | CNS Metastatic Lesions (28) | Unspecified Lung Cancer (28) | Lung Cancer (28) | Brain (26): Leptomeninges (26), Spinal Cord (2) | NA;NA | 3; NA |
| 12 | *Aghadiuno PU et al, 1985, Nigeria*^4^ | 21/89 | NA ± NA [0- 15] | 17 M, 4 F | Headaches (NA), Visual Deficits (NA) | X-Ray (NA), EEG (NA), Ventriculography (NA), Angiography (NA), Surgical Biopsy (21) | CNS Metastatic Lesions (21) | Neuroblastoma (11), Burkitt Lymphoma (9), Myeloid Leukemia (1) | Abdomen (10), Olfactory Bulb (1) | Brain (21): Leptomeninges (1), Cerebrum (9) | Chemotherapy (9); NA | NA;NA |
| 13 | *Ekpe E et al, 2019, Kenya^9^* | 6/125 | 49.2 ± 13.5 [21-79] | 6 F | NA | MRI (6), CT (6) | CNS metastatic lesions (6) | Breast Carcinoma (6) | Breast (6) | Brain (6) | Hormone Therapy (3), Trastuzumab IT (3), Chemotherapy (NA), RT (NA); NA | NA;NA |
| 14 | *Olasode BJ et al, 2000, Nigeria*^23^ | 48/210 | NA ± NA [0-64] | 16 M, 32 F | NA | NA | CNS metastatic lesions (48) | Choriocarcinoma (11), Burkitt lymphoma (10), Bronchogenic carcinoma (7), Breast carcinoma (4), Embryonal rhabdomyosarcoma (3), Unspecified Eye tumor (3), Undifferentiated carcinoma (2), Retinoblastoma (1), Primary liver cell carcinoma (1), Malignant melanoma (1), Carcinoma of the ovary (1), Rectal carcinoma (1), Thyroid carcinoma (1), Esophageal carcinoma (1), Yolk sac tumor (1) | Lung (7), Breast (4), Eye (4), Liver (1), Skin (1), Ovary (1), Rectum (1), Thyroid (1), Esophagus (1) | Brain (48) | NA;NA | NA; NA |
| 15 | *Ibrahim H et al, 2019, Nigeria*^12^ | 52/52 | 44.7 ± NA [21-71] | 52 F | Headache, nausea, and visual impairment (16), Headache and cognitive changes (14), Headache, vomiting, and seizure (14) Headache and motor deficit (8) | CT (41), MRI (11) | CNS metastatic lesion (52), > 3 Lesions (37), 3 Lesions (9), 2 Lesions (6) | Invasive ductal (32), Invasive lobular (6), Metaplastic (3), Mucinous (5), Other (6) | Breast (52) | Brain (52) | Conservative Management (52); Malaise/fatigue (27), Fever (8), Ear tinnitus (4), Alopecia (35) | NA;NA |
| 16 | *Stagno V et al, 2014, Uganda*^27^ | 8/172 | NA | NA | Visible or palpable scalp/skull or orbital mass (8) | Surgical Biopsy (8) | NA | Burkitt Lymphoma (4), Large cell B lymphoma (2), Hodgkin’s lymphoma (1), Non-Hodgkin’s lymphoma (1) | Lymphoma (8) | Brain (8) | NA;NA | NA;NA |
| 17 | *Adeloye A et al, 1976, Nigeria*^2^ | 52/213 | NA ± NA [0.6-72] | 35 M, 17 F | Hemiparesis (6), Mental changes (4), Cranial nerve palsies (4), Seizures (NA), Changes in consciousness (NA) | NA | NA | Choriocarcinoma (17), Neuroblastoma (8), Retinoblastoma (2), Rhabdomyosarcoma (1) | Uterus (17), Adrenal gland (6) Mediastinum(1) Olfactory bulb (1)), Breast (3) Kidney (3) Thyroid (2) Pancreas (2) Liver (2) Gallbladder (2) Rectum (2) Nasopharynx (2) Unknown (2) Bronchus (1) Lung (1) Stomach (1) Ovary (1) | Brain (52): Leptomeninges (6) | NA;NA | NA;NA |
| 18 | *Chikani MC et al, 2020, Nigeria*^6^ | 3/29 | NA | NA | NA | CT (3), MRI (3) | NA | NA | NA | Epidural Space (3) | Unspecified surgery (3); NA | NA;NA |
| 19 | *Mwongeli M et al, 2023, Kenya*^14^ | 15/131 | NA | NA | NA | NA | NA | NA | Breast (15) | Brain (15) | NA;NA | NA;NA |
| 20 | *Osinde TA et al, 2016, Nigeria^25^* | 30/30 | 43.5 [16-70] | 25 M, 5 F | Headache (14) | NA | NA | Unspecified Breast Cancer (23), Unspecified colon cancer (1), Unspecified Endometrial Cancer (1), Unspecified Pancreatic Cancer (1), Lymphoma (2), Other (1) | Breast (23), Lung (1), Colon (1), Endometrium (1), Pancreas (1), Paranasal Sinus (1) | Brain (30) | Whole Brain Radiotherapy (30), 30Gy in 10# (2), 30Gy in 15# (2), 15Gy in 7# (4), 28Gy in 5# (1),  20Gy in 5# (1) ; N/A | NA; 12 |
| 21 | *Adewuyi SA DA et al, 2006, Nigeria*^3^ | 5/72 | NA | NA | NA | CT (5), MRI (5) | NA | Unspecified prostate cancer (5) | Prostate (5) | Brain (5) | NA;NA | NA;NA |
| 22 | *Nesrine M et al, 2017, Tunisia*^15^ | 41/1400 | 41 ± NA [NA- NA] | 41 F | NA | CT (23), MRI (23) | NA | Breast Carcinoma (41); HER2 Positive (9), HER2 Negative (13) | Breast (41) | Brain (41) | RT (22), Chemotherapy (13), Unspecified surgical resection (4); NA | NA; Median OS: 11 |
| 23 | *Cole G et al, 1978, South Africa*^7^ | 1/27 | NA | 1 M | Delirium (1) | Post-mortem Autopsy (1) | NA | Unspecified Lung Cancer (1) | Lungs (1) | Brain (1) | NA;NA | 1;NA |
| 24 | *Badara A et al, 2019, Sene*gal*^28^* | 20/682 | 47.5 ± NA [NA- NA] | 6 M, 14 F | Intracranial hypertension syndrome (headache, vomiting, visual blurring) (18), Motor deficit (14), Seizures (10), Cerebellar syndrome (4) | MRI (3), CT (20) | CNS metastatic lesions (20), Multiple lesions (14), Single lesion (6) | Melanoma (1), Undetermined (1), Unspecified lung cancer (10), Unspecified colorectal cancer (2), Unspecified breast cancer (6) | Lung (10), Breast (6), GI (2), Skin (1) | Brain (20): Supratentorial (14) | Unspecified surgery (20); NA | 10; NA |
| 25 | *Omon HE et al, 2021, Nigeria*^24^ | 11/115 | NA | NA | NA | NA | NA | Colonic adenocarcinoma (6), Small round cell tumor (1), Rhabdomyosarcoma (2), Squamous cell carcinoma (2) | Colon (6) | Brain (11) | NA;NA | NA;NA |
| 26 | *Konan K et al, 2018,* [*Cote d'Ivoire*](https://www.scirp.org/journal/articles.aspx?searchcode=Oncology+Department+of+the+Treichville+University+Hospital%2c+Department+of+Medicine+and+Medical+Specialties%2c+Felix+Houphou-et-Boigny+University%2c+Abidjan%2c+Cote+dIvoire&searchfield=affs&page=1&skid=0).^13^ | 41/41 | 43 ± NA [27-68] | 41 F | Headache (41), Intracranial Hypertension (21), Memory Loss (12), Balance Problems (7) | CT (139), MRI (16) | CNS metastatic lesions (41), ≤ 3 lesions (16), > 3 lesions (25) | Infiltrating ductal carcinoma (40), Micropapillary carcinoma (1) | Breast (41) | Brain (41) | NA;NA | NA;NA |
| 27 | *Benna M et al, 2018, Tunisia*^5^ | 139/139 | 54 ± NA [28-86] | NA | Focal signs (42), Headaches (40), Seizures (22) | CT (139), MRI (16) | NA | Adenocarcinoma (29), squamous cell carcinoma (10), Undefined subtype (8), Small cell carcinoma (15); Unspecified Breast Cancer (50) | Lung (62), Breast (50), Colorectal (7), Skin (2), Others (13), Unknown primary (5) | Brain (139) | Chemotherapy (6); NA | NA;NA |
| 28 | *Danjuma S et al, 2022, Nigeria^8^* | 6/39 | 49.8 ± 11.8 [28-68] | 2 M, 4 F | Headache (31), Gait abnormalities (17), Limb weakness (15), Loss of vision (14), Seizure (12), Memory loss (7) | NA | NA | Unspecified Breast Cancer (4), Unspecified Prostate Cancer (2) | Breast (4), Prostate (2) | Brain (6) | Chemotherapy (6); NA | NA;NA |

**Abbreviations:**

CNS: Central Nervous System

CT: Computed Tomography

EEG: Electroencephalography

ER: Estrogen Receptor

Gy: Gray

HER2: Human Epidermal growth factor Receptor 2

MRI: Magnetic Resonance Imaging

NA: Not Available

M: Male

F: Female

OS: Overall Survival

RT: Radiotherapy

WBRT: Whole Brain Radiotherapy

**References:**

1. Adegboyega B, Joseph A, Alabi A, Omomila J, Ngema LM, Ainsworth V, et al: Patient reported outcomes following whole brain radiotherapy in patients with brain metastases in NSIA-LUTH Cancer Center. **BMC Cancer 23:**1233, 2023

2. Adeloye A, Odeku EL, Williams AO: Metastatic tumours of the brain and its enveloping structures in Ibadan, Nigeria. **Afr J Med Med Sci 5:**181-184, 1976

3. Adewuyi SA, Mbibu NH, Samaila MO, Ketiku KK, Durosinmi-Etti FA: Clinico-pathologic characterisation of metastatic prostate cancer in the Radiotherapy and Oncology Department, Ahmadu Bello University Teaching Hospital, Zaria-Nigeria: 2006-2009. **Niger Postgrad Med J 20:**45-51, 2013

4. Aghadiuno PU, Adeloye A, Olumide AA, Nottidge VA: Intracranial neoplasms in children in Ibadan, Nigeria. **Childs Nerv Syst 1:**39-44, 1985

5. Benna M, Mejri N, Mabrouk M, El Benna H, Labidi S, Daoud N, et al: Brain metastases epidemiology in a Tunisian population: trends and outcome. **CNS Oncol 7:**35-39, 2018

6. Chikani MC, Messi M, Okwunodulu O, Mezue W, Ohaegbulam S, Ndubuisi C, et al: Pattern of presentation and surgical management of spine tumors in Southeast Nigeria over a 10-year period. **Niger J Clin Pract 23:**1167-1171, 2020

7. Cole G: Intracranial space-occupying masses in mental hospital patients: necropsy study. **J Neurol Neurosurg Psychiatry 41:**730-736, 1978

8. Danjuma S, Dauda HA, Kene AI, Akau KS, Jinjiri IN: Profile and Outcome of Management of Brain Tumours in Kaduna Northwestern Nigeria. **J Korean Neurosurg Soc 65:**751-757, 2022

9. Ekpe E, Shaikh AJ, Shah J, Jacobson JS, Sayed S: Metastatic Breast Cancer in Kenya: Presentation, Pathologic Characteristics, and Patterns-Findings From a Tertiary Cancer Center. **J Glob Oncol 5:**1-11, 2019

10. Hudson DA, Krige JE, Strover RM, King HS: Malignant melanoma of the external ear. **Br J Plast Surg 43:**608-611, 1990

11. Ibebuike K, Ouma J, Gopal R: Meningiomas among intracranial neoplasms in Johannesburg, South Africa: prevalence, clinical observations and review of the literature. **Afr Health Sci 13:**118-121, 2013

12. Ibrahim H, Yaroko AA: Palliative external beam radiotherapy for advanced breast cancer patients with brain metastasis in the university college hospital Ibadan. **Ann Afr Med 18:**127-131, 2019

13. Kouassi KKY, Touré M, Seka EN, Kimso O, Oseni MA, Odo BA, et al: Profile of Patients with Breast Cancer Brain Metastasis in Abidjan. **Scientific Research Publishing 7:**201-210 2018

14. Matheka M, Mutebi M, Sayed S, Shah J, Shaikh AJ: Metastatic breast cancer in Kenya: survival, prognosis and management at a tertiary referral centre. **Ecancermedicalscience 17:**1566, 2023

15. Mejri N, Benna M, El Benna H, Soumaya L, Afrit M, Zouari B, et al: First site of recurrence after breast cancer adjuvant treatment in the era of multimodality therapy: which imaging for which patient during follow-up? **Breast Dis 37:**123-132, 2018

16. Moodley M, Budhram S, Connolly C: Profile of mortality among women with gestational trophoblastic disease infected with the human immunodeficiency virus (HIV): argument for a new poor prognostic factor. **Int J Gynecol Cancer 19:**289-293, 2009

17. Mushonga M, Nyakabau AM, Ndlovu N, Iyer HS, Bellon JR, Kanda C, et al: Patterns of Palliative Radiotherapy Utilization for Patients With Metastatic Breast Cancer in Harare, Zimbabwe. **JCO Glob Oncol 7:**1212-1219, 2021

18. Ndlovu B, Sattar MOA, Mkhaliphi MM, Leola K, Mpanza MN, Ouma JR, et al: Supraorbital eyebrow approach: A single-center experience. **Surg Neurol Int 13:**566, 2022

19. Ndubuisi CA, Ohaegbulam SC, Iroegbu LU, Ekuma ME, Mezue WC, Erechukwu UA: Histologically Confirmed Intracranial Tumors Managed at Enugu, Nigeria. **J Neurosci Rural Pract 8:**585-590, 2017

20. Nzeangung BA, Biwole ME, Kadia BM, Bechem NN, Dimala CA, Sone AM: Evolutionary aspects of non-metastatic breast cancer after primary treatment in a sub-Saharan African setting: a 16-year retrospective review at the Douala general hospital, Cameroon. **BMC Cancer 18:**32, 2018

21. Ogbole GI, Adeyinka OA, Okolo CA, Ogun AO, Atalabi OM: Low field MR imaging of sellar and parasellar lesions: experience in a developing country hospital. **Eur J Radiol 81:**e139-146, 2012

22. Ogunbiyi JO: Lung cancer at the University College Hospital, Ibadan, Nigeria. **East Afr Med J 72:**271-275, 1995

23. Olasode BJ, Shokunbi MT, Aghadiuno PU: Intracranial neoplasms in Ibadan, Nigeria. **East Afr Med J 77:**4-8, 2000

24. Omon HE, Komolafe EO, Olasode BJ, Ogunbameru R, Adefidipe AA, Anele CO, et al: Clinicopathological Profile of Central Nervous System Tumors in a Tertiary Hospital in Southwest Nigeria. **J West Afr Coll Surg 11:**1-5, 2021

25. Osinde TA, Adamu A, Jimeta JD, Chukwuocha IC: Palliative care in patients who receive whole brain radiotherapy for brain metastases in Ahmadu Bello University Teaching Hospital, Zaria. **Niger J Med 25:**215-219, 2016

26. Sherief ST, Mulatu DG, Wu F, O'Banion J, Dimaras H: Clinicopathological Presentation of Retinoblastoma in Ethiopia. **Ocul Oncol Pathol 8:**168-174, 2022

27. Stagno V, Mugamba J, Ssenyonga P, Kaaya BN, Warf BC: Presentation, pathology, and treatment outcome of brain tumors in 172 consecutive children at CURE Children's Hospital of Uganda. The predominance of the visible diagnosis and the uncertainties of epidemiology in sub-Saharan Africa. **Childs Nerv Syst 30:**137-146, 2014

28. Thiam AB, Mbaye M, Thioub M, Kala RGB, Sy EHCN, Faye M, et al: Brain Metastases: Epidemiological, Clinical, Diagnosis, Treatment and Outcome Features in Dakar. **Scientific Research Publishing 9**, 2019
