# Supplementary material for: Presentation, management, and outcomes of central nervous system metastases in Africa: Systematic review and meta-analysis
Source: Neurooncol Adv. 2024 Dec 11;7(1):vdae219. doi: 10.1093/noajnl/vdae219 (PMC11805695; doi:10.1093/noajnl/vdae219)
Supplement: vdae219_suppl_Supplementary_Table_S2 [file vdae219_suppl_supplementary_table_s2.docx]

**Supplemental Table 2.** Demographics, pre-clinical characteristics, management strategies and outcomes of all case reports included in the systematic review.

| **#** | ***Author, Year, Country*** | **Age (Years)** | **Sex** | **Non-CNS Signs and Symptoms** | **CNS Signs and Symptoms** | **Imaging Modality** | **Diagnostic Findings** | **Pathological Findings** | **Primary**  **Cancer** | **Metastasis Locations** | **Management** | **FU (Months); Outcome** |
| --- | --- | --- | --- | --- | --- | --- | --- | --- | --- | --- | --- | --- |
| 1 | *Rwomushana et al., 1975, Uganda*^1^ | 40 | F | Inguinal Node Enlargement, Heavy Vaginal Bleeding | Headache, Motor Symptoms, Sensory Symptoms, Cranial Nerve Palsy, GU Symptoms, Visual deficits, Altered Consciousness, Weight loss, Pain, Changes in Reflexes, Changes in Power, Nausea and Vomiting, Behavioral and Cognitive Deficits | CT, Angiogram,  X-ray,  Histopathology,  Labs | Tumors in Brain, Lungs, Bone, and Lymph Nodes | Kaposi Sarcoma | Skin | Supratentorial: Cerebrum, Infratentorial: Cerebellum, Hilar and Inguinal Lymph Nodes | Conservative (Chemotherapy, Radiotherapy, Antimetabolite, and Steroids)  Surgical (Craniotomy + Partial Resection and Piecemeal Resection) | NA; Death |
| 2 | *Davey et al., 1979, South Africa*^2^ | 23 | F | Heavy Vaginal Bleeding | GU Symptoms | X-ray,  Histopathology,  Labs | NA | Choriocarcinoma | Ovary | Cerebrum | Conservative (Chemotherapy, Antimetabolite, and Combination Therapy) | NA; NA |
| 3 | *Jean et al., 2021, West Africa^3^* | 5.6 | F | Abdominal Mass | Metastatic Specific Symptoms | CT | Tumors in Brain | Wilms Tumor | Kidney | Brain and Lungs | Surgical in only 3 patients (Nephrectomy) | 0.5; Death |

**Abbreviations:**

FU: Follow-up

NA: Not Available

CT: Computed Tomography

MRI: Magnetic Resonance Imaging

GU: Genitourinary

M: Male

F: Female

**References:**

**1.** Rwomushana RJ, Bailey IC, Kyalwazi SK. Kaposi's sarcoma of the brain. A case report with necropsy findings. *Cancer.* 1975; 36(3):1127-1131.

**2.** Davey DA, Fray R. Choriocarcinoma and invasive mole. A review of 10 years' experience. *S Afr Med J.* 1979; 56(22):924-931.

**3.** Jean S, Georges ADJ, Ines YDM, et al. The Management of Wilms Tumor in West Africa: A Case Series Report from the Military Teaching Hospital of Cotonou. *SAS Journal of Surgery.* 2021; 7(02):102-106.
